# Mass spectrometry imaging for spatially resolved multi-omics molecular mapping

**DOI:** 10.1038/s44303-024-00025-3

**Published:** 2024-07-17

**Authors:** Hua Zhang, Kelly H. Lu, Malik Ebbini, Penghsuan Huang, Haiyan Lu, Lingjun Li

**Affiliations:** 1 School of Pharmacy, University of Wisconsin-Madison, Madison, WI 53705 USA; 2 Department of Chemistry, University of Wisconsin-Madison, Madison, WI 53706 USA; 3 Lachman Institute for Pharmaceutical Development, School of Pharmacy, University of Wisconsin-Madison, Madison, WI 53705 USA; 4 Wisconsin Center for NanoBioSystems, School of Pharmacy, University of Wisconsin-Madison, Madison, WI 53705 USA

**Keywords:** Biophysical methods, Imaging, Analytical chemistry, Biochemistry, Chemical biology

## Abstract

The recent upswing in the integration of spatial multi-omics for conducting multidimensional information measurements is opening a new chapter in biological research. Mapping the landscape of various biomolecules including metabolites, proteins, nucleic acids, etc., and even deciphering their functional interactions and pathways is believed to provide a more holistic and nuanced exploration of the molecular intricacies within living systems. Mass spectrometry imaging (MSI) stands as a forefront technique for spatially mapping the metabolome, lipidome, and proteome within diverse tissue and cell samples. In this review, we offer a systematic survey delineating different MSI techniques for spatially resolved multi-omics analysis, elucidating their principles, capabilities, and limitations. Particularly, we focus on the advancements in methodologies aimed at augmenting the molecular sensitivity and specificity of MSI; and depict the burgeoning integration of MSI-based spatial metabolomics, lipidomics, and proteomics, encompassing the synergy with other imaging modalities. Furthermore, we offer speculative insights into the potential trajectory of MSI technology in the future.

## Introduction

**Fig. 1:**
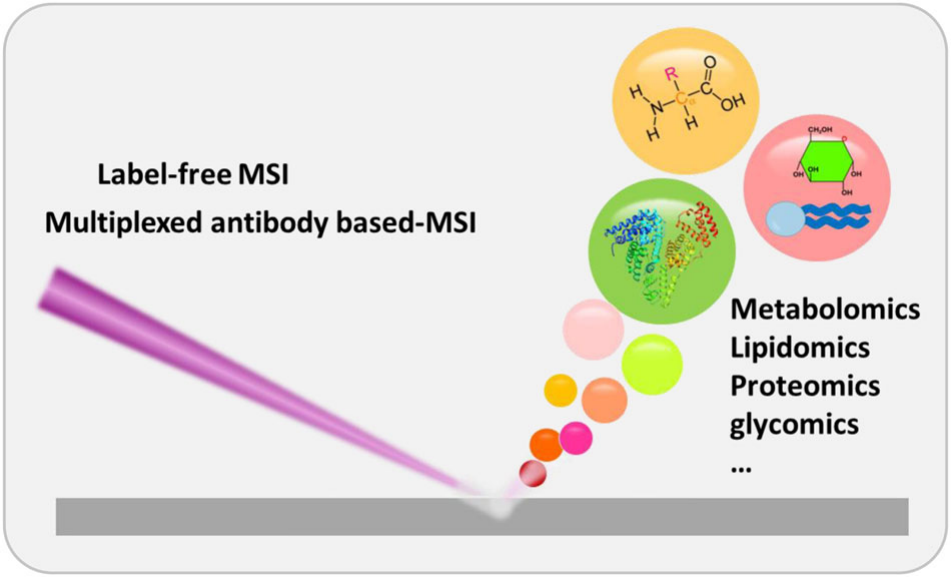
Mass spectrometry imaging (MSI) serves as a leading tool for the spatial mapping of metabolome, lipidome, and proteome.

Nearly all physiological functions of living organisms rely on the spatially organized arrangements of various biomolecules such as metabolites, proteins, and nucleic acids. Although we are familiar with examining the spatial distribution of the metabolites, proteins, nucleic acids, etc. from tissue and cell samples, our focus often leans towards one specific omics, such as metabolomics, proteomics, and transcriptomics, leading to a limited understanding of the connections across these diverse biomolecules. The present surge in integrating multi-omics approaches for conducting multidimensional spatial molecular measurements is ushering in a new era in biological research^1^. This trend marks a significant leap forward, as spatially resolved multi-omics molecular mapping would offer unprecedented insights into the intricate landscape, spatial organization, interaction, and remodeling of diverse biomolecules within biological systems^1–3^. The integration of imaging techniques has become an integral component of multi-omics mapping, propelled by significant advancements in both instrumentation and methodologies across spatial genomics, transcriptomics, proteomics, and metabolomics^1^. Deep sequencing has been intensively employed for spatial genomics and transcriptomics, providing detailed information about the genetic and transcriptomic compositions of the tissue samples^4,5^. In particular, mass spectrometry imaging (MSI) serves as a leading modality for the spatial mapping of metabolome, lipidome, and proteome owing to its exceptionally informative nature, superior sensitivity, and high spatial resolution (Fig. 1)^6,7^. The advances in spatial genomics and transcriptomics have been comprehensively reviewed elsewhere^2,8,9^ and are beyond the scope of this review. Herein we focus on the emerging advancements and future insights of MSI for spatially resolved metabolomics, lipidomics, and proteomics over the recent years.

Advancements in ion optics and innovative ionization strategies have significantly pushed forward MSI capabilities, allowing for spatial resolutions to reach several micrometers and even down to the nanometer level^6^. Currently, secondary ion mass spectrometry (SIMS) imaging can provide micrometer to nanometer scale lateral spatial resolution, and a 5-micrometer lateral spatial resolution is now available for commercial matrix-assisted laser desorption/ionization (MALDI) MS platform. Enhancements in spatial resolution have empowered researchers to achieve success in conducting MSI at a single-cell resolution for the exploration of cell-to-cell heterogeneity^10,11^. In addition to the hardware advances, many exciting advances in sample preparation and handling also remarkably enhanced MSI capabilities. For example, emerging on-tissue chemical derivatization strategies have been adopted in MSI, which enhances the sensitivity, specificity, and coverage for specific types of biomolecules^12–14^. Moreover, the integration of ion mobility (IM) with MSI has provided a distinctive capability for effectively separating isomeric compounds within tissue samples^15–18^. As hardware and software advancements persist, MSI is embracing high-spatial resolution 3-dimensional (3D) renderings of biological samples, marking promising frontiers such as constructing comprehensive molecular 3D atlases for tissue samples and potentially entire organisms^19–21^. With all the continuous advancements, MSI is poised to offer more comprehensive data for spatially resolved metabolomics, lipidomic, and proteomics, facilitating a better understanding of various physiological and pathological processes, such as tissue development and cancer heterogeneity.

In this review, we first provide an overview of MSI and various MSI methods to offer a basic understanding of the technology. Then we discuss the recent advancements and innovative applications of MSI in the spatially resolved metabolomics, lipidomics, and proteomics. Particularly, we delve into the latest advances of MSI including enzymatic-assisted multimodal imaging, integrations of chemical derivatizations, and ion mobility separation for improved molecular coverage. We outline the challenges of MSI in these emerging application areas and engage in discussions about potential solutions to address them. Finally, we offer speculative insights into the potential trajectory of this burgeoning technology in the future.

## Overview of mass spectrometry imaging method

**Fig. 2:**
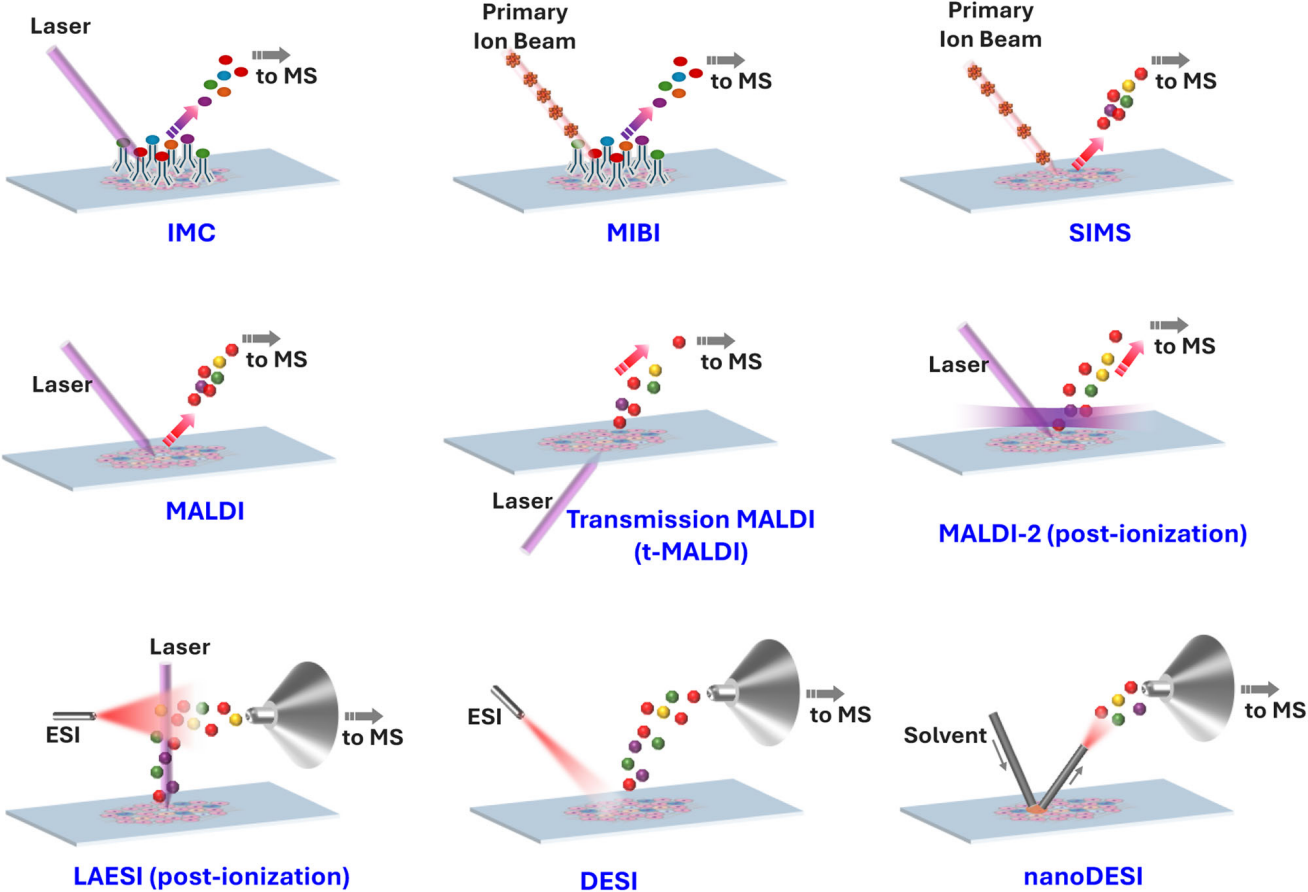
Representative MS imaging methods. Graphical representation of the main techniques for MS imaging, including multiplexed antibody-based and label-free MSI methods. Inverted Y-shaped symbols, antibodies; colored balls, tags on antibodies or analyte ions; horizontal laser beam in MALDI-2 technique; ESI electrospray ionization. Modified from figure adapted with permission from ref. ^6^

The general setup of MSI is raster sampling across the tissue section following a pre-defined (x,y) grid, in which the biomolecules within each pixel are ionized for mass spectrometry (MS) interrogation and a mass spectrum is generated for each pixel. With the mass spectrum of each single pixel and its corresponding positional information, a MS image of the tissue sample is generated by building a heat map based on the signal intensity of a given mass-to-charge ratio (*m/z* value). Consequently, spatial distribution information for the molecule is obtained. Usually, the size of the pixel determines the spatial resolution of the MS image; the smaller the pixel size, the higher spatial resolution for MSI. Diverse MS imaging approaches have been established for spatially resolved proteomics and metabolomics. Depending on the sample preparation strategies, these imaging approaches can be broadly categorized into two categories: label-free MS imaging and antibody-based MS imaging (Fig. 2).

### Label-free MS imaging

Label-free MSI capitalizes on the intrinsic advantages of MS, allowing the imaging of thousands of molecules in a single experiment without prior knowledge or the need of labels or antibodies assistance for detection. Label-free MSI has been extensively applied to the mapping of small metabolites, lipids, peptides, glycans, proteins, etc. in tissue samples. Representative MSI techniques in this regime include MALDI^22–25^, SIMS^26,27^, and desorption electrospray ionization (DESI)^28,29^.

#### MALDI

MALDI is among the most widely adopted MS imaging tools; it enables soft ionization of diverse endogenous metabolites, lipids, peptides, glycans, and even proteins directly from the tissue, providing unprecedented molecular information for the elucidation of molecular landscape of tissue sample^30,31^. In MALDI-MSI, the tissue section is coated with a matrix compound via nebulization or sublimation, then scanned with a laser beam in a grid pattern, resulting in desorption and ionization of biomolecules from different locations. The MALDI matrix, often a small organic compound, is essential for MALDI-MSI; the matrix assists in the ionization process by absorbing laser energy, promoting the desorption and ionization of analytes. The choice of matrix is crucial as it can affect the detection sensitivity, spatial resolution, and types of molecules that can be ionized efficiently during MALDI-MSI experiments. In addition to conventional MALDI matrices like α-cyano-4-hydroxycinnamic acid (CHCA), 2,5-dihydroxy benzoic acid (DHB), and 3,5-dimethoxy-4-hydroxycinnamic acid (sinapic acid), a variety of novel matrices have been developed for MALDI-MS imaging with enhanced sensitivity and molecule coverage^32–35^. Moreover, the optics system in MALDI-MS imaging is undergoing rapid development to achieve higher spatial resolution and improved ionization efficiency. For instance, a novel optics system was coupled with atmospheric pressure MALDI to enable lateral resolution of 1.4 μm^22^, which could be further reduced to 600 nm for brain tissue via transmission-mode MALDI and laser post-ionization (t-MALID-2)^23^. It is important to note that in laser post-ionization (MALDI-2), a secondary ionization source, often a beam of high-energy photons or electrons, is used to further ionize molecules in the sample plume that was initially generated by the traditional MALDI laser^36^. This secondary ionization step enhances the ionization efficiency and sensitivity of certain compounds that have low ionization yields from the traditional MALDI laser. Recent studies have showcased the ability of MALDI-2 to bring forth a remarkable sensitivity improvement for metabolites such as steroids and phosphatidylethanolamine, cholesterol, glucosyl ceramide^17,36,37^. Currently, MALDI-MS imaging with a lateral resolution of 5–10 μm is available on some commercial instruments. Recent techniques such as Space M^25^ and MicroMS^24^ have enabled the detection of abundant metabolites from large-scale single human cell population, providing meaningful insights into the mystery of single-cell heterogeneity. This availability together with its robust ionization performance, have established MALDI as an effective technique for spatially resolved metabolome and lipidome analysis.

#### SIMS

SIMS is a surface analysis technique that provides detailed information about the elemental and molecular composition of materials. Similar to MALDI, SIMS operates under vacuum. However, instead of a laser ionization source, SIMS emits energetic particles from a finely focused primary ion beam that ablate and penetrate into the upper surface layers, inducing a short-lived collision cascade with atoms and molecules as a consequence of energy dissipation. This subsequently leads to ejection of secondary ions from the sample. The secondary ions carrying the information of analytes are detected by the mass analyzer^38,39^. Only a small fraction (~1%) of the sputtered population exists in charged state, and this number is highly influenced by the applied electric field, the mass of the colliding partners, the dissipating energy of primary ion impact, and the angle of the primary ion relative to the surface normal^39,40^. NanoSIMS, utilizing a highly focused primary beam with reactive ions (such as Cs^+^ or O^−^), enables nanometer-scale spatial resolution and high mass accuracy without sacrificing sensitivity^41–43^. The SIMS technique can be broadly classified into two areas, static SIMS and dynamic SIMS. Static SIMS is often employed in surface analysis, capturing analytes from the outermost layer, and primarily used in qualitative molecular imaging. On the other hand, dynamic SIMS is more suitable for depth analysis, as ablation is transmitted through multiple layers by using a higher dose primary beam, and is mainly used in qualitative elemental imaging^44^. The popularity of SIMS stems partially from the ability to detect all elements with low detection limit, high sensitivity, wide dynamic range, and minimal sample preparation^45,46^. Recent efforts, such as modifying instrument configuration^47–49^ and introducing antibody-assisted imaging^50–53^, have further enhanced the spatial resolution and multiplexity. Though SIMS has been able to perform MS imaging with lateral resolution down to 20–50 nm^50,54,55^ and maintains the highest spatial resolution for MS imaging, SIMS is a “hard” ionization technique and is unable to ionize most peptides or proteins. Furthermore, SIMS-MS instruments are usually prohibitively expensive which limits its widespread application for MS imaging. While the high energy of the ion beam in SIMS limits the acquisition of intact biomolecules from the tissue samples, extensive efforts have been made to couple SIMS with softer desorption/ionization processes, using novel ion sources such as (CO_2_)_n_^+^/Ar_n_^+^ gas clusters^49^ and (H_2_O)_n_^+^ clusters^56^.

#### DESI

Unlike techniques such as MALDI and SIMS that typically operate under vacuum conditions, DESI is an ambient ionization technique^28^. DESI-MS imaging allows for the direct analysis of samples in their native state under atmospheric conditions, without the need for extensive sample preparation. This characteristic makes DESI-MS imaging particularly useful for the rapid mapping of metabolites and proteins in tissue samples without compromising their native properties. In DESI-MS imaging, a pneumatically assisted electrospray emitter is used to generate a stream of charged solvent droplets, which is directed at the sample surface, causing the extraction and desorption of molecules on the tissue surface. Highly charged secondary microdroplets carrying the dissolved analyte molecules are formed from the kinetic impact of the primary droplets, leading to the ionization of biomolecules before being sent into the MS inlet for analysis^28,29^. The spatial resolution achieved in DESI MS imaging usually ranges between 150 and 200 μm^28,29^, nonetheless, a spatial resolution of 35 μm has been attained through meticulous optimization of experimental conditions^57^. To further improve the spatial resolution, a variant of DESI, termed as nanospray desorption electrospray ionization (nano-DESI)^58^, has been developed later, pushing forward the spatial resolution down to 7–10 μm for direct imaging of proteoforms in biological tissues^59,60^. In nano-DESI MS imaging, a capillary system creates a tiny liquid bridge between two capillaries, generating a solvent stream that contacts the sample surface. This contact facilitates the desorption and extraction of analyte molecules from the surface, and these analyte molecules are then transferred for ionization and analyzed by a mass spectrometer. It is worth mentioning that the choice of solvent plays a crucial role in DESI and nano-DESI MS imaging; the solvent influences the efficiency of desorption and ionization of molecules from the sample surface, and the spray stability. For improving the accuracy and sensitivity of MS imaging for specific types of biomolecules, internal standards, and chemical derivatization reagents can be added into the solvent. For instance, rose bengal was added in the nano-DESI solvent for online photochemical derivatization of carbon-carbon double bonds for the imaging of isomeric unsaturated lipids in mouse brain tissues^61^. While DESI-MSI or nano-DESI is primarily applied for spatially resolved lipidomics and metabolomics, its capability to analyze native proteins directly from tissue sections is emerging as a promising area for its application^18,60,62,63^. This is owing to the unique advantage of DESI and nano-DESI, which can easily generate multiply-charged protein ions directly from the tissue samples. By combining top-down MS approach, intact proteins on the tissue can be desorbed, ionized, and then directly fragmented into smaller peptides, allowing for the characterization of intact proteins, including their sequence, modifications, and isoforms^18,60^. Based on DESI, an air-flow-assisted desorption electrospray ionization (AFADESI) method was proposed for whole-body molecular imaging^64^; a key difference between the two methods is that after the sample undergoes ionization via an electrospray plume, ions will be transported over long distances by an airflow for MSI in AFADESI^65^.

#### Other label-free MS imaging methods

Indeed, label-free MS imaging provides a direct approach to mapping the biomolecules across single cells and various tissue samples. In addition to the aforementioned methods, other label-free methods have been developed for MS imaging of small biomolecules. These methods have significantly advanced the capability of MS imaging, especially for the improvement of spatial resolution and acquisition of chemical information.

One example would be laser desorption ionization (LDI)^66^. In contrast to MALDI, LDI operates with the absence of matrix. With the assistance of microlensed fiber^21^ or using an extreme ultraviolet laser source^67^, a lateral resolution down to 75 nm can be achieved in LDI, which allows the MS imaging of drug distribution within single cells. Similar to MALDI that uses organic chemicals to assist analyte ionization, surface assisted laser desorption ionization^68^ uses nanostructured substrates instead of conventional organic matrices. The nanosubstrates play the same role as the conventional organic matrices that absorb the laser energy to aid the desorption and ionization of analytes^69^. Nanosubstrates are advantageous for small molecule analysis as they generate fewer interference peaks in the low *m/z* range than conventional organic matrices^70^. Another advantage of nanosubstrates is the ability to functionalize their surface with ligands that bind specifically to analytes of interest, thus leading to enhanced signal intensity for the analytes of interest^68^.

Laser ablation electrospray ionization (LAESI)^71^ tailors toward improvement of the sensitivity for biomolecules. In this method, the sample is ablated with a focused laser pulse under atmospheric conditions, resulting in a plume with ablated biomolecules, clusters, and particulate matters. After that occurs, an electrospray hits the ablated biomolecules with charged droplets. The biomolecules coalesce with the charged droplets, leading to ionization of the biomolecules^72^.

As another MSI technology, liquid extraction surface analysis (LESA)^73^ allows the experimentalists to select a solvent that can dissolve the analytes of interest. A pipette tip held above the tissue section dispenses a small volume of the selected solvent. After that occurs, a liquid microjunction forms between the pipette tip and the tissue section. The analytes of interest are then dissolved in the liquid microjunction and get sampled by the pipette tip. As a result, a tissue extract solution is obtained for an MS experiment. If this process is repeated at each point on the tissue, a tissue extract solution can be obtained at every “pixel”, thus enabling MS imaging^74^. One disadvantage of this approach is the poor spatial resolution; the spatial resolution of LESA MSI is around 1 mm, which is poor in comparison to the spatial resolution of MALDI MSI (~10 µm)^75^. However, LESA MSI has a major advantage: the significantly larger pixel size means a significantly larger amount of sample is available for ionization and detection, thus leading to enhanced sensitivity^73^.

### Multiplexed antibody-based MS imaging

While label-free MS imaging remains the most straightforward approach for mapping metabolites, lipids, and proteins across various tissue and cell samples, a significant challenge persists in achieving the necessary sensitivity for direct in situ imaging of large biomolecules, such as high-molecular-weight proteins. To achieve a higher sensitivity for large biomolecules, a multiplexed antibody-based MS imaging strategy has been developed. The multiplexed antibody-based MS imaging strategy is a combination of immunohistochemistry and MS imaging, by utilizing antigen-specific antibodies to recognize the target analytes such as proteins. The antibodies carry unique mass-tags that can be used to generate signature reporter ions when sampled by a laser or ion beam. The specificity of this antibody-mass-tag labeling approach is generally more favorable for confidently identifying and mapping the protein composition within cells or tissue samples. Antibody-based MS imaging technologies, including multiplex ion beam imaging (MIBI)^50,52^ and imaging mass cytometry (IMC)^76–78^, have been developed for MS imaging of proteins within the tissue samples at high-resolution. Both technologies utilize unique metal isotopes (e.g., isotopically pure lanthanide metals) tagged antibodies to label proteins on tissue but differ in their metal tag ionization methods. MIBI is based on high-resolution nanoscale SIMS, enabling determination of up to 100 targets simultaneously with a spatial resolution down to 260 nm^52^. Whereas the IMC, a technology developed from CyTOF mass cytometry^79^, utilized a high-resolution laser ablation system together with a low-dispersion laser ablation chamber, which could image 36 proteins simultaneously at a lateral resolution of 1 μm^80^. Given the excellent spatial resolution, both MIBI and IMC have been employed for spatial mapping of protein composition in tumor tissues with subcellular resolution, revealing remarkable insights about tumor microenvironments and heterogeneity^51,52,80–82^.

In addition to metal isotope labeling methodologies, organic mass tag-labeled antibodies were adopted for multiplexed immunohistochemical MS imaging as well^83–85^. For instance, there is a photocleavable mass tag based MSI approach introduced by Thiery et al. termed TArgeted Multiplex Mass Spectrometry IMaging (TAMSIM)^84,86^. In this work, a mass tag is designed to contain a trityl group, a linker, and an NHS ester for antibody conjugation. The trityl group is activated by absorbing a UV light at 355 nm, resulting in cleaving the C-S bond and leaving a carbocation on the mass tag. The positive charge is stabilized by the trityl group, and the reporter is readily analyzed in MALDI. The multiplicity of the TAMSIM mass tag is achieved by modifying the hydrocarbon chain length on the trityl group, yielding a mass difference of 14 Da increment between each tag^84^. Moreover, sequential analysis with MSI and immunofluorescent microscopy (IFM) imaging can be done by selectively integrating a fluorophore to the antibody in the synthesis step, providing a platform for IHC staining validation and quality control as well as quantitation by correlating IFM and MSI intensities. Recently, a new form of reporter ion has been introduced by Yagnik and coworkers, combining MALDI-MSI with IHC, termed MALDI-IHC^83^. This photocleavable mass tag (PC-MT) probe utilizes peptide sequence as reporter ion, where the reporter tag is attached to the antibody via a photocleavable linker that is cleaved under UV exposure, releasing the peptide reporter for MS detection. Unlike the trityl mass tags, PC-MT probes release only the peptide reporter ion but leave the bulky linker on the antibody, avoiding any further in-source fragmentations. One drawback of this approach is that the inherent difference in the proton affinity of the reporter peptide sequence plays an important role in controlling the ionization efficiency, potentially leading to a biased analysis. However, the versatility of the PC-MT probe has been demonstrated by multiple applications. For example, FFPE breast cancer tissues were stained with 19 antibody probes while two were dual labeled for IFM, and the resulting MSI and IFM images show almost identical morphology^83,87^. In addition to the development of photocleavable mass tags, another recent development was an eight multiplexing antibody labeled boronic mass tag (BMT) designed for DESI MSI under atmospheric pressure^85^. The BMT tag is activated by the hydrolysis of the boronic-ester bond caused by the electrospray microdroplets during DESI sampling under positive mode.

Targeted antibody-labeling-assisted MSI has substantially overcome the difficulties posed by conventional IHC microscopy imaging techniques, offering advantages such as the absence of autofluorescence background signal, a log fold increase in dynamic range, no spectral overlap, and high multiplicity. High multiplicity can be achieved because the reporter mass can be resolved even with a low-resolution instrument. The function of linkers is essential in the process of releasing photoactivated reporter ions. Depending on the instrument and linker design, a linker can be cleaved either through an external UV source or the MS ionization source. An ideal reporter ion should possess a high ionization efficiency, the capability for multiplex mass tuning with similar synthesis method, and a *m/z* outside of the matrix peak region if matrix application is required. Lastly, the choice of an antibody probe is crucial for an unbiased experiment, where false positive or false negative imaging results could be observed due to nonspecific binding or poor antibody-epitope recognition, respectively, highlighting the need for cross-validation methods. Antibody-assisted MSI is a targeted approach; the selection of antibody is highly dictated by the protein targets. To expand this strategy beyond proteomic studies, other probes can be introduced to label a wider spectrum of analytes. For example, lectins, the carbohydrate binding probes, can be conjugated to the PC-MT for glycomics analysis without enzymatic digestion, preserving the native localization of glycans residing on intact proteins and lipids^87^. Oligonucleotide probes can hybridize to their complementary mRNA/DNA sequences, enabling multiplexed DNA/RNA analysis including gene expression monitoring, genetic profiling, and pathogen detection^88,89^. The challenges associated with antibody-labeling MSI are (1) low labeling efficiency due to low antibody-antigen binding affinity, (2) high cost of commercially available antibodies, (3) the boundaries of this approach are defined due to limited synthesizable antibodies, and (4) it is necessary to know the analyte(s) of interest before the start of the experiment, making this imaging approach incompatible with the discovery of unknown biomolecules. Furthermore, while these mass tag-based antibody approaches provide reliable relative quantitation, detection of the reporter ions rather than the protein itself implies complete loss of the molecular and structural information of the bound proteins. An increasing number of studies have demonstrated that the perturbation of protein structure and dysregulation of protein posttranslational modification (PTM) are vitally important to understanding pathological mechanisms. For this aspect, it is imperative to explore the spatially resolved proteome in a manner that provides structural and compositional information.

## Tandem MS identification for MS imaging

After MSI data is acquired through label-free or multiplexed antibody-based methods, it is possible to identify the molecules of interest by accurate mass matching. In accurate mass matching, the observed intact mass of a molecule is matched to the mass of a known molecule in a database within a certain mass error tolerance range^90,91^. However, there is a problem with this approach: a single MS scan will not be able to discriminate isobaric or isomeric species, thus leading to two or more known molecules in the database appearing to match one observed peak. This is especially problematic in experiments where the mass accuracy and mass resolution are low, making isobaric species even more difficult to distinguish based on accurate mass matching. Given the problem with isobaric or isomeric species, accurate mass matching is not always sufficient for confident identification. As a result, tandem MS (MS/MS or MS^n^) is needed in some situations.

The convention is to perform MS/MS using a tissue section adjacent to the tissue section used for MS imaging^92^, but the identifications are more definitive if MS/MS and MS imaging are performed on the same tissue section. For instance, Dilillo et al.^93^ experimented to perform LC-MS/MS and MALDI MS imaging on the same tissue section. Similarly, Randall et al.^94^ demonstrated LESA extraction and MALDI MS imaging on the same tissue section; as LESA extraction can be used for LC-MS/MS, the implication is that LC-MS/MS data and MALDI MS imaging data can be obtained using a single tissue sample. It is noted that care must be taken to ensure that the solvent used for extraction does not cause significant analyte delocalization and that there is no severe loss in MALDI signal intensity resulting from LESA extraction.

Another way to obtain more direct evidence for identification is by collecting the MS/MS data through the same experimental setup as the MS imaging data. However, this is difficult to achieve in the context of proteins in MALDI MS imaging, as MALDI generates primarily singly charged ions and those singly charged protein ions usually have poor fragmentation efficiency^92^. As a result, in some cases it may not be possible to do MALDI MS/MS. Investigators have addressed this issue by employing an indirect identification strategy through LC-MS/MS in an adjacent tissue section^92^. It is important to note that there is a drawback associated with this approach: if the MALDI MS imaging data is lacking in terms of mass accuracy and mass resolution, it is not possible to match the observed MALDI *m/z* values to the observed LC-MS/MS precursor *m/z* values. In order to address this issue, a MALDI instrument with high resolving power and mass accuracy can be used, such as a FTICR instrument^95^. On the other hand, it is relatively easy to collect the MS/MS data with DESI and nano-DESI through the same experimental setup as the MS imaging data. DESI is a liquid extraction based ambient ionization method that can generate multiply charged protein ions^96^. For example, Yang et al.^96^ generated nano-DESI MS imaging data and nano-DESI MS/MS data in rodent brain tissue sections. Other investigators have also successfully performed nano-DESI MS/MS alongside nano-DESI MS imaging^60,62,97^.

The previously cited examples of MS/MS data were collected without chemical derivatization, but it is important to note that in some cases MS/MS cannot provide the desired information in the absence of chemical derivatization. For example, lipids have substantial structural complexity containing double bond positional isomers, cis/trans isomers, and sn-positional isomers. In the absence of chemical derivatization, it is oftentimes impossible to distinguish these structural isomers solely with MS/MS. To address this issue, investigators have developed on-tissue chemical derivatization strategies coupled with MS/MS. For instance, peracetic acid assisted on-tissue epoxidation of unsaturated fatty acids was developed for profiling fatty acid double bond positional isomers in the mouse tumor tissues^98^. Another on-tissue chemical derivatization method developed by Sun et al.^99^ uses methyltrioxorhenium (MTO)-catalyzed epoxidation of double bonds (within lipids) with a urea hydrogen peroxide (UHP)/hexafluoroisopropanol (HFIP) system. To further improve the MS/MS throughput in chemical derivatization strategies assisted MSI, Guo et al.^100^ utilized on-tissue Paternò–Büchi (PB) derivatization and a miniature dual-LIT mass spectrometer for high throughput MS/MS imaging of phospholipid isomers. This method enabled 10 MS/MS experiments in a single scan and the distinction between 20 phospholipid double bond positional isomers with high sensitivity.

It is worth mentioning that there are some situations where MS/MS is not sufficient for the confident identification of some molecules due to inadequate information, thus necessitating the use of MS^3^ or MS^n^. In MS^n^, the fragment ions from MS^2^ can undergo further fragmentation, which yields more characteristic fragments. For instance, Guo et al. combined MS^3^ imaging and on-tissue photochemical derivatization to distinguish phospholipid double bond positional isomers and phospholipid sn-positional isomers^101^. Similarly, Mavroudakis et al.^102^ distinguished between prostaglandin isomers through MS^3^ and silver ion adduction to enhance the detection sensitivity for prostaglandins. Specifically, they were able to distinguish between PGE_2_, PGD_2_, and Δ12-PGD_2_ in a mouse uterus implantation site. MS^3^ has also been used to distinguish between synthetic cannabinoid isomers^103^ and steroid structural isomers^104^.

Multiple reaction monitoring (MRM), also known as selective reaction monitoring, is another tool for tandem MS identification in MSI^105^. MRM can be used for the confident identification of multiple compounds in MS imaging. For example, Weigand et al.^106^ demonstrated the power of MRM in MS imaging using nanoDESI-MSI on a triple quadrupole instrument with MRM acquisition mode to detect lipids in mouse brain and rat kidney. With this strategy, they were able to distinguish isobaric phospholipids and different eicosanoid isomers. It is important to note that coupling MRM in MSI offers a platform to profile targeted analytes with low abundance in the tissue samples^107,108^.

Another useful tool for identification would be data dependent acquisition (DDA), in which the most highly abundant precursor ions are selected for fragmentation^109^. Ellis et al.^110^. published a method to enable automated acquisition of DDA MS/MS imaging data for lipids in parallel with high resolution MS imaging data. This method enabled the identification, validation, and localization of 104 lipids in rat cerebellar tissue. DDA is useful, but it suffers from a major disadvantage: the low abundance molecules will be masked, as only the high abundance molecules are selected for fragmentation. To address this issue, investigators have used data independent acquisition (DIA), where all the molecules within a given *m/z* window are selected for fragmentation and the process is repeated for every *m/z* window within the *m/z* range^109^. While DIA has been used for LC-MS/MS^109^, it is rarely applied in MSI so far. If DIA MS/MS imaging is performed in the future, that would certainly lead to highly complex data, thus necessitating more powerful data analysis tools. Even with DDA MS/MS imaging, which is less complicated than DIA, there is still a need for new data analysis tools. There have been ongoing efforts to develop pipelines such as MSIpixel^111^ and those of the SIMSEF project^112^ for DDA MS/MS imaging data analysis. Indeed, as imaging data continues to become more complex, the sophistication of the data analysis methods will need to adjust accordingly.

## Data analysis

Multi-omics MS imaging data can be acquired through the use of label-free methods or multiplexed antibody-based methods, but data acquisition is only the first step. Following data acquisition, data analysis is crucial for extracting meaningful biological information from MSI datasets. Data analysis of MSI involves processing and interpreting the MSI datasets to visualize the spatial distribution of biomolecules across the samples, as well as extracting relevant biological information from the MSI results. The high complexity and substantial size of the MSI datasets stems from the retention of multi-dimensional information, encompassing spatial information, the mass spectrum associated with each pixel, and, in certain cases, additional ion mobility information. Particularly, an increase in spatial resolution leads to an exponential expansion of the data file size. Thus, powerful data analysis platforms are demanded for MSI. A detailed review of MSI data analysis has been given elsewhere^32,113,114^, here we focus on the recent advancements in MSI data analysis.

### Data analysis software

Commercially available software packages can be used for MS imaging data analysis, but there are also free software options^32^. One widely used data analysis platform is MSiReader, which was created with MATLAB^115^. MSiReader contains vendor agnostic data analysis tools that can be used for more sophisticated MSI data, including quantification and co-registration with other imaging modalities^116^. In addition, an increasing number of data analysis software applications are being developed based on open-source programming languages. For example, Cardinal, a software package created with R, integrates statistical modeling^117^ and segmentation^118^. BASIS is a python-based platform that utilizes pattern recognition and machine learning to perform comparative analysis for large sample numbers^119^, and the built-in bioinformatics tools are intended to robustly scale to fit the needs of sizable clinical cohorts.

### Network analysis

Network analysis is used to study the relationships between various biomolecules within a biological sample. The network analysis has been adopted in MSI data interpretation. For instance, Dong et al.^120^ developed a new strategy called iMS2Net (imaging MS dataset-sourced multiscale network) to study metabolic responses from an environmental pollutant (PM2.5) in a suitable mouse fetal model, offering variation and covariation information within/between organs to external stimuli. The iMS2Net generated network analysis results revealed associations between metabolites and inflammatory cytokines upon exposure, demonstrating the potential for incorporating network analysis in multi-omics research. Other investigators have also performed network analysis through existing data analysis tools, such as MetaboAnalyst and SCiLS Lab software^121,122^.

### Artificial intelligence assisted MS imaging data analysis

**Fig. 3:**
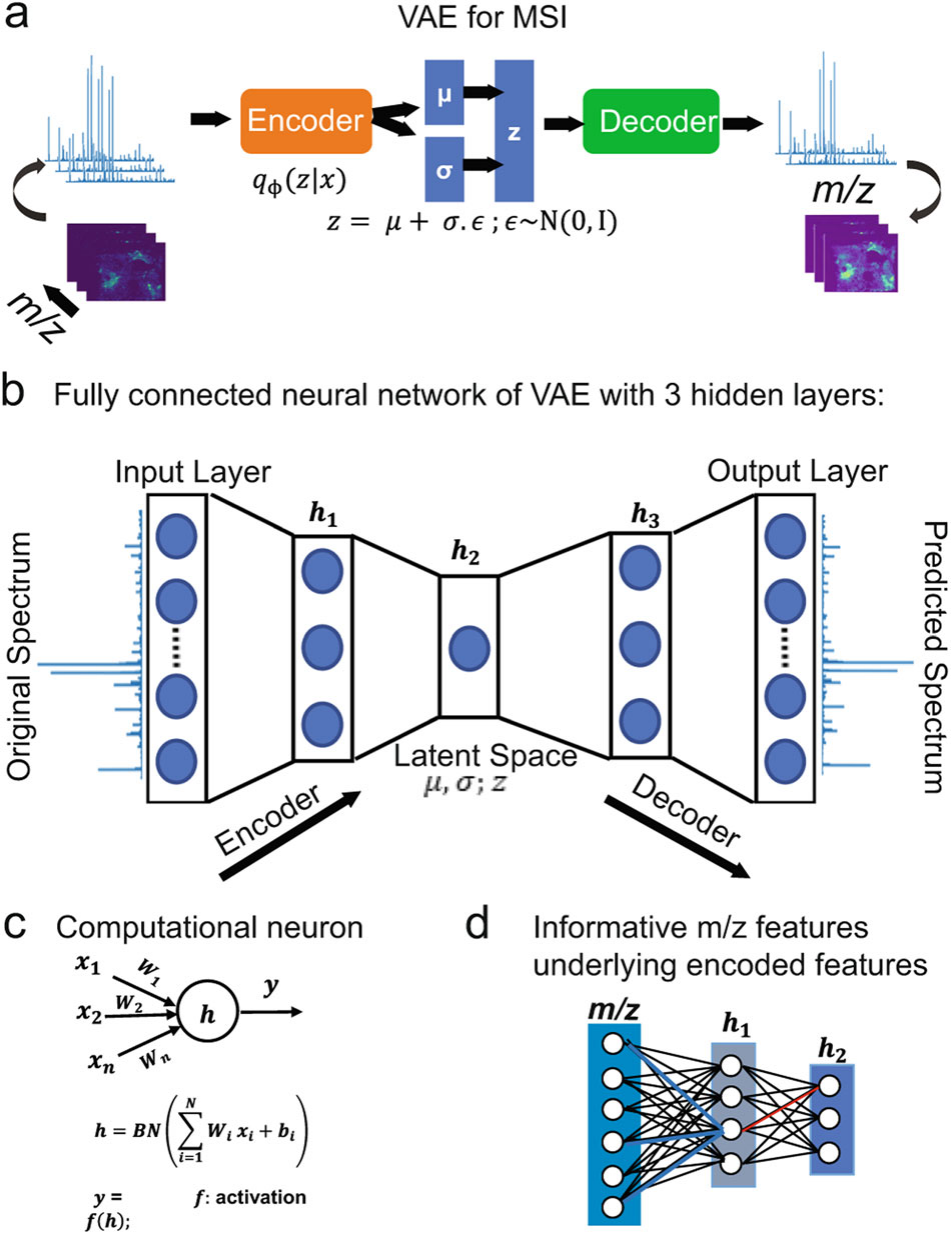
Neural-network architecture of variational autoencoder for mass spectrometry imaging data analysis. **a** Visual representation of variational autoencoder for MS imaging. Spectra on left represent experimental spectra, while spectra on right represent predicted spectra that come after dimensionality reduction resulting from the variational autoencoder. **b** Visual representation of five layers in fully connected neural network. In between those two layers, three hidden layers in the fully connected neural network. **c** Batch normalization for neural network regularization. **d** Statistical analysis on the neural-network weight parameter was used to pinpoint useful *m/z* features. Used under the terms of the Creative Commons Attribution 4.0 International License^124^. Copyright 2021, The Authors, published by Springer Nature.

The artificial intelligence (AI) methods used for interpreting MS data are emerging. Disease relevant biomarkers have been found using algorithms such as least absolute shrinkage and selection operator (LASSO), hierarchical cluster analysis (HCA), self-organizing map (SOM), random forest (RF), and support vector machine^123^. Additionally, hidden layer artificial neural networks, deep learning convolutional neural networks, and deep neural networks have been mentioned in handling large data volume^123^. Particularly, AI methods are increasingly employed for the analysis of MS imaging data^124–126^. For example, Abdelmoula et al.^124^ developed a deep learning data analysis strategy known as msiPL (MSI Peak Learning) based on a variational autoencoder neural network (Fig. 3). The variational autoencoder reduces the data dimensionality without inducing significant loss of essential spectral features. It is noted that MS imaging data sets suffer from the “curse of dimensionality”—the presence of many mass spectra with many peaks in each spectrum will inevitably lead to large and complex data, which decreases the efficiency of data analysis work. In order to address this dilemma, the msiPL could reduce data dimensionality even with simple clustering algorithm (Gaussian Mixture Modeling)^127^. Moreover, the Uniform Manifold Approximation and Projection method^128,129^ and the Hierarchical Stochastic Neighbor Embedding (HSNE) method are two well-known nonlinear data reduction algorithms in terms of addressing limitations posed by memory requirement and CPU/GPU time^130^.

Delineating the spatial distribution of multiple analytes using co-localization and clustering is an essential feature in multi-omics analysis. In addition to conventional tools, recent advances with AI technology have thrived, offering more comprehensive insights from biological samples. For example, Ovchinnikova et al.^125^ developed a machine learning approach, ColocML, to explore the co-localization performance, which found that the best performing co-localization measures were the semi-supervised deep learning Pi model and the cosine score applied after median thresholding. One of the limitations from conventional clustering methods is they are dependent on a specific mathematical hypothesis about the regions of interest (ROIs), constrains the identification of ROIs that does not agree with the assumption, which is especially problematic for samples with high heterogeneity. In response to this challenge, Guo et al.^126^ developed a deep learning strategy for clustering, namely divide-and-conquer (dc)-DeepMSI, which utilizes a flexible unsupervised deep learning model for metabolic heterogeneity analysis from MSI data without prior knowledge of histology. Based on specific modes of spat-contig and spat-spor, dc-DeepMSI can be used to identify either spatially contiguous ROIs or spatially sporadic ROIs. Clearly, there is a need for a flexible clustering algorithm that can better account for highly heterogeneous tissue.

MS imaging research applies cutting-edge AI methods^124^. Indeed, the traditional AI methods cannot efficiently perform data analysis due to the large size and high complexity of MS imaging data^131,132^. Not only does the use of cutting-edge AI methods indicate a bright future for MS imaging data analysis, but there is also an increase in publicly available data that is making it easier for computational work to be done. For example, Oetjen et al.^133^ provided benchmark datasets to the whole MS imaging community with the intent of promoting bioinformatics research and developing advanced computational solutions to address the challenges associated with MSI data analysis. All of the benchmark datasets were provided in the imzML format, making it easily accessible to MSI community^134^. The datasets were shared in the MetaboLights and *GigaScience* GigaDB repository. Since more researchers have access to repositories with publicly available data, more progress in MS imaging data analysis is expected to take place in the years to come.

Together, the bright future of multi-omics MS imaging is further reinforced by advancements in data processing and interpretation. As more information can be obtained, powerful software tools to facilitate the handling and bridging the biological relevance from large data files are in high demand. MS imaging for spatial multi-omics requires multiple experiments, thus giving rise to multiple MS imaging omics datasets. It is crucial to accurately pipeline all the information from one omics dataset to another in a pixel-positional manner, bringing forth one integrated dataset with all the desired multi-omics information. This will guarantee the information crosstalk between different omics is accurate and clear after co-registration.

## Improvement for MSI for spatially resolved molecular mapping

As discussed earlier, label-free MSI emerges as an efficient and straightforward approach for exploratory studies in biochemistry and biomedicine. A substantial amount of information can be obtained from a single experiment. However, due to the inherent complexity and heterogeneity of tissue and cell samples, certain biomolecules pose challenges for detection using conventional MSI techniques. In particular, biomolecules with low abundance or high molecular weight experience diminished ionization efficiency. Relying on a conventional MSI mode is inadequate for capturing a comprehensive snapshot of all analytes, and distinguishing isomeric species is impossible without orthogonal separation methods. In response to these challenges, we describe recent technological and methodological advancements to enhance molecular coverage, sensitivity, specificity, and measurement accuracy in MSI applications.

### Enzymatic-assisted multimodal MS imaging

The inherent soft ionization of MALDI MSI poses challenges for some large biomolecules. This soft ionization tends to generate primarily singly charged ions that surpass the optimal MALDI mass range and lack sufficient ionization energy to release molecules from the sample surface^135–137^. To address these issues, different enzymes are employed to assist the detection of previously unattainable large biomolecules and enhance the sensitivity of low-abundance analytes. For example, the commonly used endopeptidase trypsin is widely employed in bottom-up proteomic MSI, and it digests proteins by cleaving the C-terminal after lysine or arginine. The fragmented peptides are extracted for MSI, and the identification and distribution of the original parent proteins are analyzed through accurate mass matching with database searching engines^92,138,139^. Furthermore, the combination of more than one enzyme treatment and sequential MSI allows for obtaining multimodal information. Glycomics and proteomics imaging involve releasing N-glycans using PNGase F (peptide N-glycosidase F) followed by tryptic peptide MSI^140^. The localization of large extracellular matrix components is characterized using collagenase and elastase to target collagens and elastin, which are not cleavable with trypsin^141^. Characterization of the extracellular matrisome is achieved by sequential enzymatic digestions including chondroitinase ABC, PNGase F, elastase, and collagenase III for chondroitin sulfated glycosaminoglycans, N-glycans, elastin, and collagens analysis, respectively^142^. In all the aforementioned studies, a consistent finding is that a higher protein sequence coverage and more confident peptide identification can be achieved when using multiple enzymatic treatments. This is likely due to more complete and comprehensive digestion, as a result of higher target exposure to enzymes after removing other components^143,144^. This concept can be applied to improve the sensitivity of proteins with surface modifications, such as glycans, by performing deglycosylation to remove unwanted features, making the target analytes more accessible during the protease treatments^143^. Additionally, enzymatic digestions can reduce the molecular weight of large molecules with a constant mass shift, enabling the detection of target analytes within the optimal MALDI mass range^145^. Given the vast versatility and diversity of enzyme functions and their potential combinations, novel enzymatic approaches can be developed and applied to specifically enhance the sensitivity of low-abundance, large, and neutral molecules. Moreover, these approaches contribute to a more comprehensive understanding of post-translational modifications, offering in-depth insights into molecular localization and biochemical information.

### chemical derivatizations

Despite the continuous improvements for MS instrumentation, a persistent challenge of low detection sensitivity for certain compounds remains significant during MS imaging. Factors such as low abundance of analytes, weak ionization efficiency, ionization suppression, and endogenous interferences contribute to visualization problems for specific molecules. To address these challenges, on-tissue chemical derivatization is emerging as a promising solution for an enhanced visualization capability for MS imaging platforms. Through the utilization of chemical tagging on a specific moiety of the target analytes, on-tissue chemical derivatization not only boosts ionization efficiency but also adeptly eliminates analytes from regions prone to potential isobaric interferences. Additionally, it can introduce a differentiation factor in tandem MS for isomeric biomolecules, further enhancing the precision and specificity of the MSI results. By integrating on-tissue chemical derivatization and MS imaging, emerging studies have demonstrated a significant enhancement in detection sensitivity and specificity for various biomolecules such as neurotransmitters^146^, carboxyl-containing metabolites^147^, primary and secondary hydroxyl-containing metabolites^148^, N-glycans^149–151^, lipids^98,152^, etc. For instance, peracetic acid was employed for on-tissue epoxidation of unsaturated fatty acids in the mouse tumor tissues, leading to the spatially resolved mapping of fatty acid isomers^98^. A more detailed review of the chemical derivatization reagents for MS imaging has been covered elsewhere^12–14^. It is important to note that the choice of on-tissue chemical derivatization method depends on the specific molecules of interest, the type of tissue being analyzed, and the overall goals of the study. Preserving the integrity of the tissues is required during the on-tissue chemical derivatization and optimization is usually required to have a complete labeling of target analytes while avoiding the delocalization of the biomolecules on the tissue samples.

### ion mobility separation

**Fig. 4:**
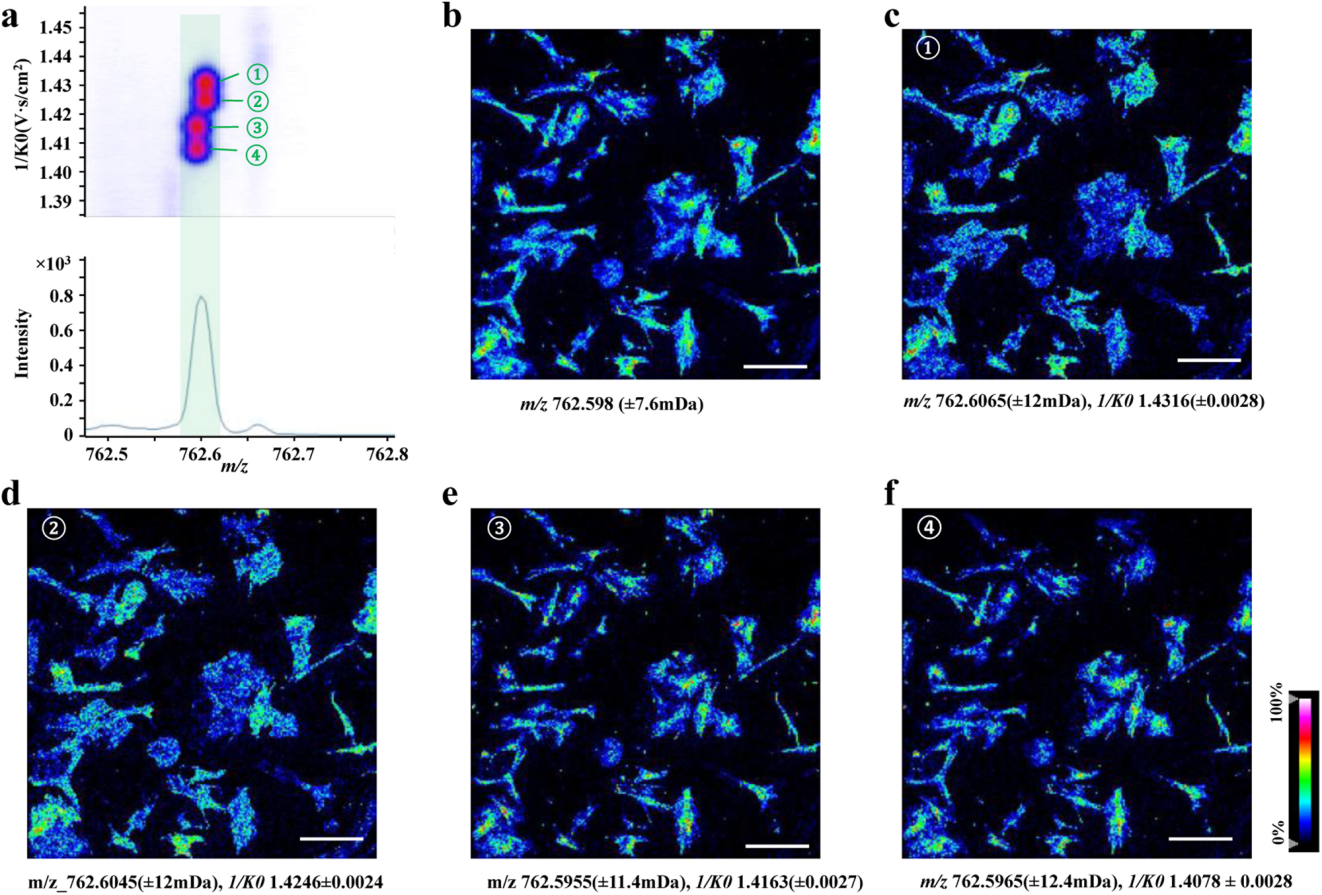
MALDI-IM-MSI of the activated human pancreatic stellate cells (PSC). **a** Representative ion-mobility MS incorporated data at a mass window of *m/z* 762.5 to 762.8, (**b**) MS images constructed based on *m/z* value alone, (**c**–**f**) MS images of isobaric and isomeric lipids revealed based on incorporation of the *m/z* value and collisional cross-section (CCS) value information. Scale bar, 400 µm. Adapted with permission from ref. ^10^

Ion mobility (IM) offers a unique gas phase separation for the biomolecule ions generated during MSI raster sampling, which adds an orthogonal analytical dimension for the recognition and separation of isobars and isomers. With the assistance of IM, collision cross sections (CCS) of each ion are calculated and the MSI images are constructed based on an integration of the *m/z* and CCS value. Commercial ion mobility modalities, such as Drift Tube Ion Mobility Spectrometry (DTIMS), Trapped Ion Mobility Spectrometry (TIMS), Traveling Wave Ion Mobility Spectrometry (TWIMS), and Field Asymmetric Waveform Ion Mobility Spectrometry (FAIMS), are increasing the accessibility of ion mobility mass spectrometry imaging (IM-MSI) within the MSI community. For example, DESI-FAIMS-MS has been established for extracting proteins directly from biological tissue sections^18^. Recently, TIMS has been integrated with MALDI-MSI and nano-DESI-MSI platforms for imaging lipid molecules in single-cell and mouse brain tissue samples, showing an enhanced specificity for recognition of lipid isobaric and isomeric species^10,16^. As shown in Fig. 4, more lipid species could be revealed from single-cell samples with the assistance of ion mobility separation while only one peak was observed when using MS alone^10^. Quadrupole-cyclic traveling wave ion mobility-time of flight mass spectrometer (Q-Cyclic IM-TOF-MS) is equipped with a DESI source for multiplex MS/MS imaging of distinct lipids from biological tissues^15^. It is worth mentioning that the on-line gas phase separation capability during IM-MSI experiments is dependent on the resolving power of IM. Currently, a resolving power of 100–200 is accessible with the cutting-edge commercial ion mobility spectrometry (IMS) instruments. To enhance resolution, one strategy involves augmenting the path length or the number of passes around the mobility device. For example, lossless ion manipulation (SLIM) devices leverage a remarkable separation path length of approximately 1094 m via 81 passes, enabling the attainment of resolving powers ~1860^153^, while in the case of cyclic ion mobility (cIM), a resolving power of 750 (with 100 transits) has been reported^154^. However, it is important to note that the sensitivity could be a challenge in these devices due to the radial loss of ions over time. An alternative approach for enhancing the separation capability in IM-MSI involves employing chemical modification for the target analytes on the tissue samples. For instance, chiral derivatizing agents (CDAs) add the separation of enantiomeric compounds by converting the enantiomers into diastereomeric derivatives via covalent labeling^155,156^.

### quantification in MS imaging

Though the MS imaging provides a powerful platform to map the spatial distribution of various molecules in different biological systems, such as lipids, small metabolites, drugs, peptides, neuropeptides, and large proteins, the robust and accurate quantification is currently impeded by numerous technical challenges. Unlike the LC-MS/MS having the front-end separation and steady ionization, the MS imaging suffers from large pixel-to-pixel variation stemming from matrix-effect, co-crystallization of analyte-matrix, selection of matrices, morphology of tissues, and variability of laser ablation or solvent extraction^157^. Extensive efforts have been made to improve the quantification performance and obtain reproducible results. Among all, stable isotope labeling (SIL) is one of the ideal analytical approaches to quantify a specific chemical in biological tissues, in which the labeled compound can be used as an internal standard to normalize the signal intensity and achieve the relative quantification. Common isotopic labeled compounds for SIL contain the synthesized ^13^C, ^15^N, and ^2^H to preserve the same chemical property and ionization efficiency but with slight mass shift from the naturally occurring one. However, this approach is not considered cost-effective, as it requires customized isotope labeling and is only applicable to the compound of interest. Instead of customized SIL technique, the non-endogenous internal standards (IS) have been developed. Class-specific IS adopt non-endogenous chemicals with near-identical ionization efficiency and known concentration to normalize the same class of endogenous chemicals in biological systems. In SIL and non-endogenous IS relative quantification approaches, the known concentration standards are sprayed uniformly across the tissue samples, and the signal intensities of endogenous chemicals can therefore be normalized to SIL or IS accordingly from pixel to pixel after the MSI acquisition. For example, Vandenbosch et al. presented a robust quantitative MSI of lipids in mouse brain tissues by applying multiclass internal standard mixture^158^.

Besides the relative quantification, many efforts have been made toward the investigation of absolute quantification in MSI as previously discussed by Kertesz et al.^159^. The high-quality and reproducibility of absolute quantification is not easily accessible, especially when acquiring high spatial resolution MSI data and handling high heterogeneity tissue samples^160^. Some improvements have been made to compensate for this drawback such as utilizing the serial frozen spiked-tissue homogenates^161^. Robust quantification results have been obtained by generation of calibration curves with standard-spiked homogenized mimetic tissue models in combination with normalizing signal intensities to evenly-distributed IS sprayed on tissue sections^162^. Although the calibration curves constructed with spiked tissue homogenate can compensate for the matrix-effect to some extent, this technique cannot comprehensively solve the problem of high heterogeneity tissue such as cancerous tissues. The tissue homogenates are average conditions of overall tissue environment, so they cannot accurately reflect the properties of specific tissue types and the chemical microenvironment of every pixel sampled in MSI^159,163^.

## Integration of multi-omics imaging

**Fig. 5:**
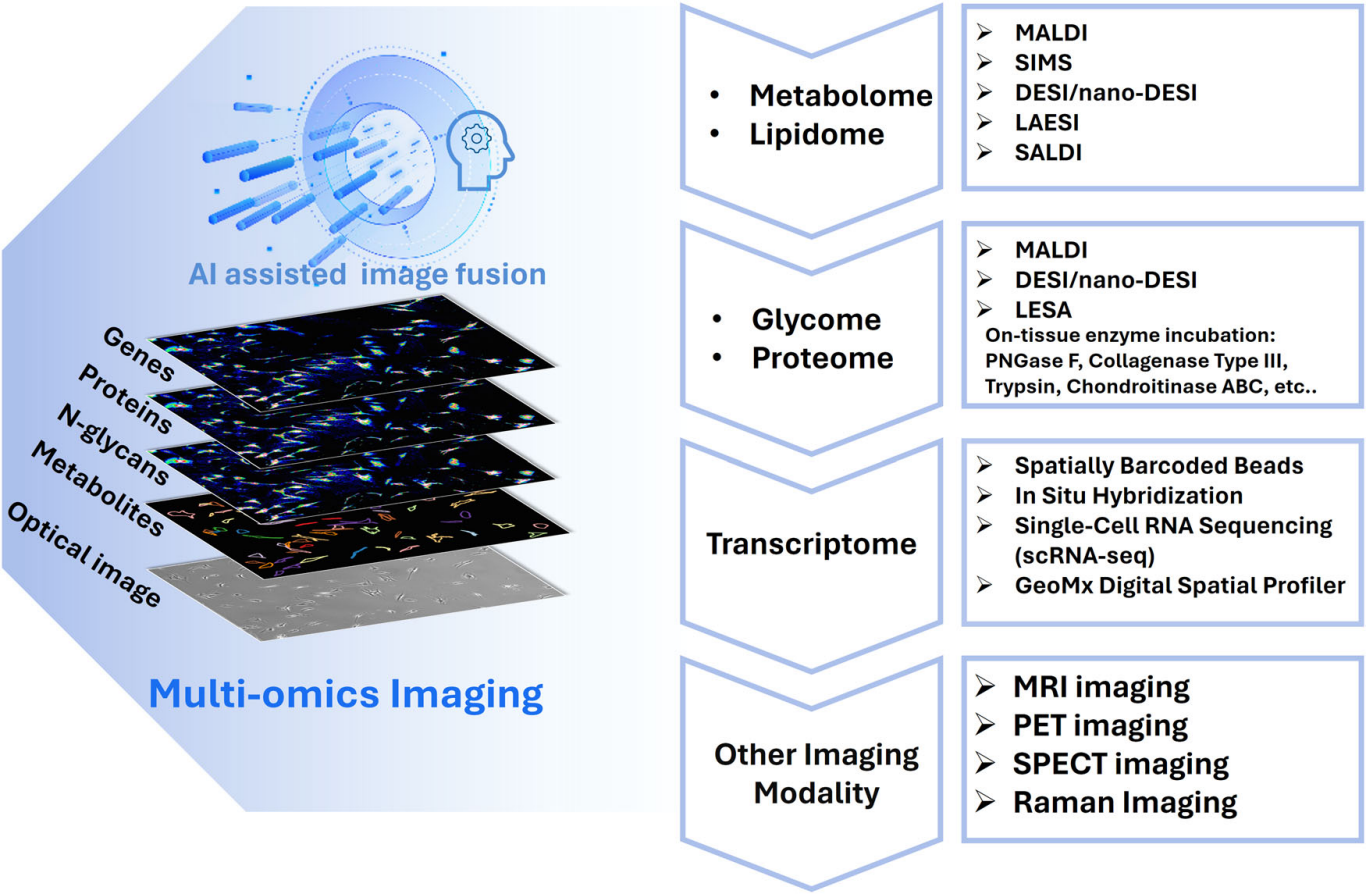
Spatially resolved multi-omics via integration of MS imaging and other imaging modalities. Integration of various omics data, such as genome, transcriptome, proteome, lipidome, and metabolome, requires accurate computational co-registration/imaging fusion of the omics data from a single tissue section or sequential tissue sections. The MSI spatial-omics dataset can be further combined with other spatial molecular profiling modalities, such as spatial transcriptomics, MRI, PET, SPECT, and Raman imaging.

Imaging has arrived in the era of multi-omics mapping owing to the major advances in instrumental and methodological developments in spatial genomics, transcriptomics, proteomics, and metabolomics (Fig. 5). Multi-omics integrates a variety of omics data such as genome, transcriptome, proteome with PTMs, lipidome, and metabolome to understand the complicated biological systems, which can further be implemented in deciphering disease mechanisms and accelerating therapeutic development^45^. Owing to the untargeted and high-throughput nature of MSI, MSI allows for mapping of different classes of biomolecules within their native spatial context in a single tissue. The combination of lipids, N-glycans, and tryptic peptides has been proposed and applied in spatial MS imaging of a single FFPE tissue^164^. The three different spatial omics data have been integrated in MALDI-MSI of a single FFPE tissue slide, and this protocol has the potential to be applied to examine more archived samples in tissue banks. As another example, a novel strategy for ToF-SIMS imaging of multiple biomolecules combines labeled (via IHC, ISH, and enzyme-based staining) and label-free approaches. This method features a tissue-friendly sample preparation and enables simultaneous observation of metabolites, lipids, peptides, proteins, and mRNA in tissues, revealing region-specific distribution patterns in the hippocampus of an AD mouse model^45^. The valuable information from sequential experiments allows the characterization of the pathological interactions of biomolecules, the change in microenvironment in unhealthy states, and the communication network of molecular markers. With all the multi-omics-based MS imaging, the crosstalk between biomolecules can be spatially mapped in tissue samples; this information would be highly valuable for discovering novel biomarkers.

**Fig. 6:**
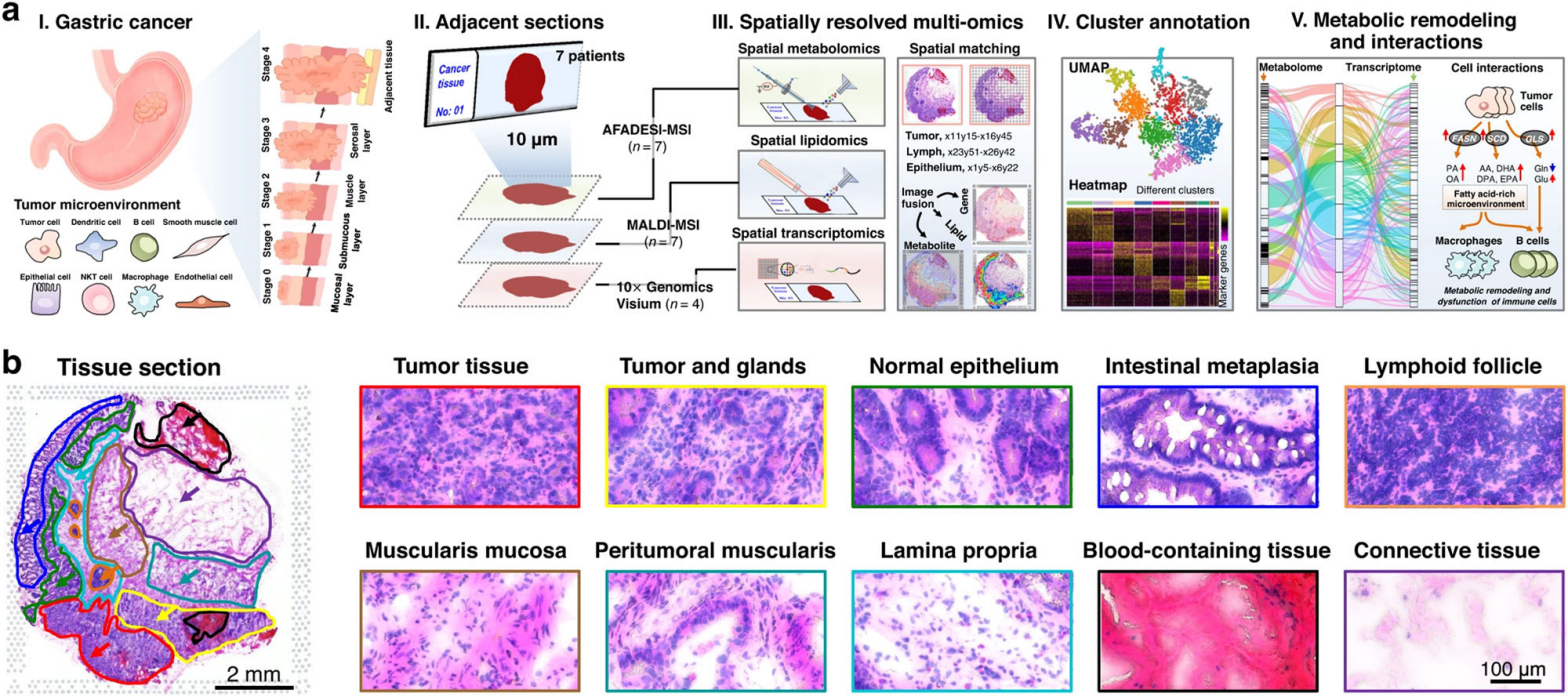
Spatially resolved multi-omics reveals intratumor heterogeneity of gastric cancer. **a** Strategy of integrated spatially resolved multi-omics for highlighting tumor metabolic remodeling and interactions. **b** H&E stain image of gastric cancer tissue section from patient and ×40 magnified H&E stain image of different gastric cancer tissue regions, scale bar = 2 mm for whole tissue section, scale bar = 100 μm for magnified images. The experiment was repeated three times. Adapted with permission from ref.^171^

Recent advances in the addition of spatially resolved genomics and transcriptomics along with peptidomics, lipidomics, and metabolomics give better cellular information in linking genotype and phenotype changes in diseases^165–168^. Spatial genomics and transcriptomics are to analyze and visualize the spatial organization of genetic material or RNA transcripts within tissues. Integrating these datasets provides a holistic view of how molecular activities vary across different regions of a tissue or cell, aiding in understanding various physiological and pathological processes, such as cancer heterogeneity, tissue development, or response to treatments. For instance, the integration of laser-assisted inductively coupled plasma MSI (LA-ICP-MSI) and MALDI has addressed the limitation of MALDI in tracing metal and metalloprotein concentrations in biological samples^169^. With a designed gold nanoparticle stabilized capsule (Au-NPSC), the distribution of a tumor necrosis factor alpha (TNF-α) protein has shown correlation with changes in lipid expression. The tissue region-specific connection between Au concentration and lipid regulation provides valuable information in delineating metal-aided biological pathways. These findings would not be observed without combining data from two MSI experiments^170^. In recent developments, an integration of spatial transcriptomes, metabolomes, and lipidomes has emerged, harmonizing histology, MSI, and spatial transcriptomics (Fig. 6)^168,171,172^. This integrated approach enables precise measurements of mRNA transcripts and low-molecular-weight metabolites across different tissue regions. For instance, He et al. showcased an integration of MSI-based spatial metabolomics and lipidomics with microarray-based spatial transcriptomics to hierarchically visualize the intratumor metabolic heterogeneity and cell metabolic interactions in the same gastric cancer sample^171^. With the multi-omics dataset, gastric tumor-associated metabolic reprogramming was visualized at metabolic-transcriptional levels; marker metabolites, lipids, and genes were connected in metabolic pathways and colocalized in the heterogeneous cancer tissues^171^.

The combination of multiple omics data reveals the complete spectrum of all the biomolecules in biological samples, which can gain valuable insights in aspects including cellular phenotype, cell-cell interactions, cellular heterogeneity, and responses to drug treatment^173^. However, establishing connections between these distinct disciplines and comprehending the intricate relationships among different molecular layers remains a pivotal challenge. Integrating data from genomics, transcriptomics, proteomics, and metabolomics in a spatially resolved manner demands sophisticated analytical frameworks capable of reconciling and overlaying diverse molecular information. This integration is essential for unraveling the complex interplay and dependencies among genes, transcripts, proteins, and metabolites within specific spatial contexts, ultimately enabling a more comprehensive understanding of biological systems and their functioning.

## Conclusion

Spatial multi-omics is an exciting area of research, as it opens new doors for understanding the intricate interrelationships among different classes of biomolecules within a biological tissue. There are multiple tools that can aid in spatial multi-omics research. MS imaging is a particularly useful tool; it can generate spatially resolved metabolomics, lipidomics, and proteomics data for systems-level integration. Indeed, existing literature has confirmed the feasibility of combining multiple omics data from MS imaging. It is anticipated that research in this field will make significant progress in the future thanks to relevant recent advancements in enzymatic assisted multimodal imaging, chemical derivatization, and ion mobility separation. The field is expected to also benefit from future improvements in instrumentation and innovation in methodology, which would lead to enhanced detection sensitivity and spatial resolution. These advancements will inevitably increase the complexity of the data, thus necessitating the development and use of more powerful AI-based data analysis tools in the future. Given that cutting-edge AI methods have already been used in MS imaging research, it is expected that significant advancements in AI-based data analysis will take place in the future. Another anticipated area of progress includes an enhanced accuracy in co-registering multi-omics data, leading to improved precision in aligning spatial information across different omics datasets. This will lead to obtaining more accurate conclusions from the data. Co-registration will also be important for integrating MS imaging data with data from other imaging modalities, such as spatial transcriptomics, magnetic resonance imaging and positron emission tomography; the combination of MS information with other imaging modalities is expected to provide more powerful biological conclusions and insights. Indeed, there is already published work that lends credence to this idea^174^. Lastly, it is anticipated that orthogonal approaches will be utilized to validate the findings from the MS imaging data. In order to perform orthogonal validation, researchers can utilize non-MS-based biological techniques, such as Western blotting, IFM, and other functional validations. By comparing the qualitative agreement between the results obtained from non-MS-based biological approaches and MS imaging, molecular mechanisms underlying biological processes can be elucidated with greater confidence, which will provide more integrated viewpoint of molecular heterogeneity at the systems and functional level.
